# Synthesis and crystal structure of calcium dizinc iron(III) tris­(orthophosphate), CaZn_2_Fe(PO_4_)_3_

**DOI:** 10.1107/S2056989016012421

**Published:** 2016-08-05

**Authors:** Jamal Khmiyas, Abderrazzak Assani, Mohamed Saadi, Lahcen El Ammari

**Affiliations:** a Laboratoire de Chimie du Solide Appliquée, Faculty of Sciences, Mohammed V University in Rabat, Avenue Ibn Battouta, BP 1014, Rabat, Morocco

**Keywords:** crystal structure, CaZn_2_Fe(PO_4_)_3_, transition metal phosphate, solid-state reaction synthesis

## Abstract

Single crystals of the title compound, CaZn_2_Fe(PO_4_)_3_, were synthesized by conventional solid-state reaction. In the asymmetric unit, all atoms are located in fully occupied general positions of the *P*2_1_/*c* space group. The zinc atoms are located on two crystallographically independent sites with tetra­hedral and distorted triangular-based bipyramidal geometries. Two edge-sharing triangular bipyramidal ZnO_5_ units form a dimer, which is linked to slightly deformed FeO_6_ octa­hedra *via* a common edge. The resulting chains are inter­connected through PO_4_ tetra­hedra to form a layer perpendicular to the *b* axis. Moreover, the remaining PO_4_ and ZnO_4_ tetra­hedra are linked together through common vertices to form tapes parallel to the *c* axis and surrounding a chain of Ca^2+^ cations to build a sheet, also perpendicular to the *b* axis. The stacking of the two layers along the *b* axis leads to the resulting three-dimensional framework, which defines channels in which the Ca^2+^ cations are located, each cation being coordinated by seven oxygen atoms.

## Abstract 2

The orthophosphate CaZn_2_Fe(PO_4_)_3_ crystallizes in the monoclinic system. The structure of this phosphate is built up from edge-sharing [ZnO_5_] polyhedra linked together by FeO_6_ octa­hedra and PO_4_ tetra­hedra.

## Chemical context   

Microporous compounds with an open anionic framework containing transition metals have been widely studied during recent years, especially iron phosphates, because of their potential applications in several fields such as gas sensing (Abdurahman *et al.*, 2014 ▸), catalysis (Ai, 1999 ▸), as cathode materials for rechargeable lithium batteries (Masquelier *et al.*, 1998 ▸), biocompatibility of glass fibres for tissue engineering (Ahmed *et al.*, 2004 ▸), and immobilization of spent nuclear fuel (Mesko & Day, 1999 ▸). Metal phosphates with an open framework can exhibit different architectures such as linear-chain, layered and three-dimensional structures with channels or cavities where a variety of cations with different sizes, ratio and charges are accommodated. The occupancy of the allowed sites by cations can provide different properties such as remarkable flexibility, fast ionic conduction and low thermal expansion, mainly observed in the compounds belonging to the NASICON family with the general formula *MM*′_2_P_3_O_12_ (where *M* = alkali metal, alkaline-earth metal or a vacant site and *M*′ = Zr, Ti, Hf, *etc*.; Senbhagaraman *et al.*, 1993 ▸). In our previous hydro­thermal investigations, a variety of compounds have been synthesized and characterized with different ratios of alkaline earth metal:P, *viz.* Sr_2_Mn_3_(HPO_4_)_2_(PO_4_)_2_ (Khmiyas *et al.*, 2013 ▸), BaMn^II^_2_Mn^III^(PO_4_)_3_ (Assani *et al.*, 2013 ▸), Mg_7_(PO_4_)_2_(HPO_4_)_4_ (Assani *et al.*, 2011 ▸). In this context, our inter­est is focused on the synthesis of new iron orthophosphates with an open-framework structure. Accordingly, we have succeeded in synthesizing and structurally characterizing a new calcium, zinc and iron-based open-framework phosphate, namely CaZn_2_Fe(PO_4_)_3_.

## Structural commentary   

**Figure 1:**
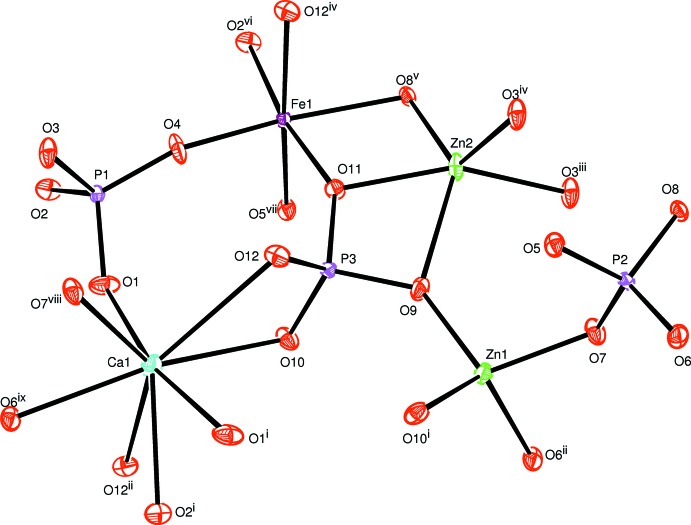
The principal building units in the structure of the title compound. Displacement ellipsoids are drawn at the 50% probability level. [Symmetry codes: (i) *x*, −*y* + 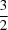, *z* − 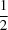; (ii) *x*, −*y* + 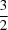, *z* + 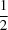; (iii) *x* + 1, *y*, *z*; (iv) −*x* + 1, −*y* + 1, −*z* + 1; (v) −*x* + 2, −*y* + 1, −*z* + 1; (vi) −*x* + 1, −*y* + 1, −*z* + 2; (vii) *x*, *y*, *z* + 1; (viii) *x* − 1, *y*, *z*; (ix) *x* − 1, −*y* + 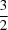, *z* + 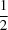.]

**Figure 2:**
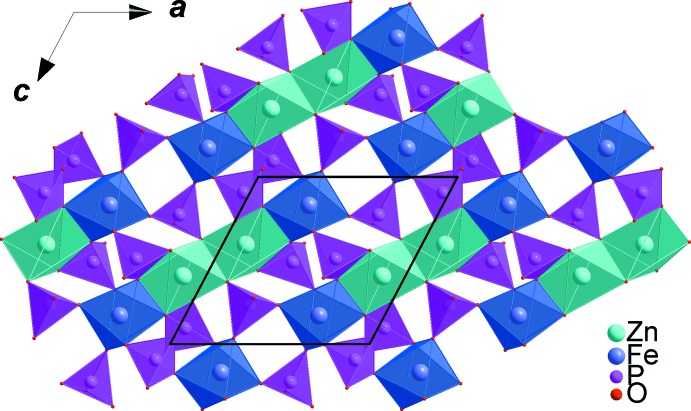
Edge-sharing triangular bipyramidal ZnO_5_ units linked to FeO_6_ octa­hedra and to PO_4_ tetra­hedra, forming a layer perpendicular to the *b* axis.

**Figure 3:**
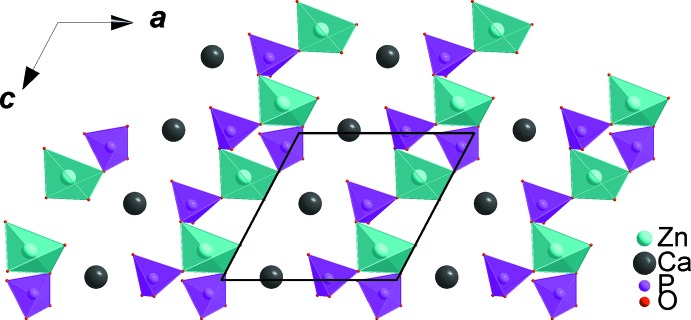
A layer perpendicular to the *b* axis, resulting from the chains connected *via* vertices of the ZnO_4_ and PO_4_ tetra­hedra.

**Figure 4:**
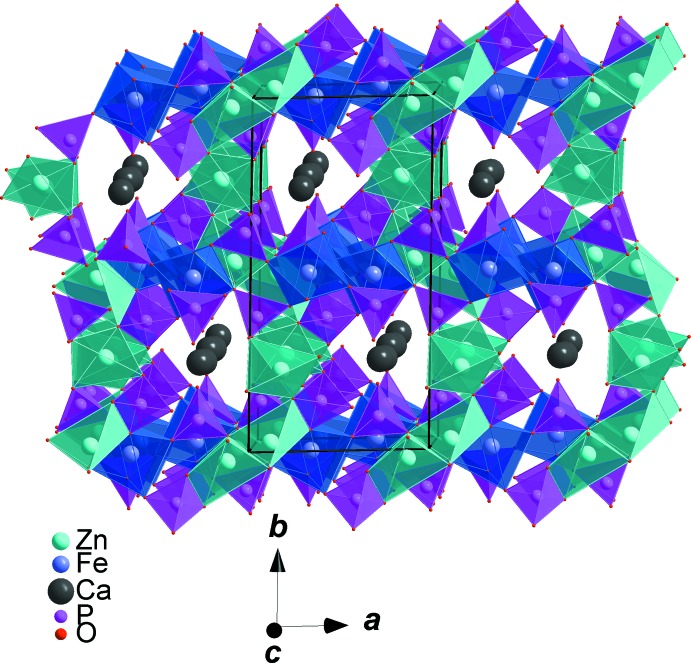
Polyhedral representation of CaZn_2_Fe(PO_4_)_3_, showing the channels running along the [001] direction.

All atoms in asymmetric unit of the title compound occupy general positions of the *P*2_1_/*c* space group. The refinement of this model was very easy and lead to an ordered structure in which the zinc cations occupy two sites with different environments. The coordination numbers of all cations were confirmed by bond-valence-sum calculations (Brown & Altermatt, 1985 ▸). The obtained values for Ca^II+^, Zn^II+^, Fe^III+^ and P^V+^ are as expected, *viz*. Ca1 (1.93), Zn1 (2.00), Zn2 (1.91), Fe1 (3.04), P1 (5.11), P2 (4.97) and P3 (4.94). The crystal structure is build up from PO_4_ and Zn1O_4_ tetra­hedra, distorted triangular-based bipyramidal Zn2O_5_ and FeO_6_ octa­hedra, as shown in Fig. 1 ▸. The FeO_6_ octa­hedra are slightly deformed with Fe—O distances varying from 1.8908 (8) to 2.1318 (8) Å and share a common edge with the highly distorted [(Zn2)_2_O_8_] dimer resulting from the edge-sharing of two triangular-based bipyramidal Zn2O_5_ units. Sequences of these polyhedra build chains inter­connected by PO_4_ tetra­hedra, forming a layer perpendicular to the *b* axis, as shown in Fig. 2 ▸. The remaining Zn1O_4_ tetra­hedra are linked to irregular PO_4_ groups *via* common corners, forming tapes parallel to the *c* axis, which are linked together by Ca^2+^ cations in sheets perpendicular to the *b* axis (see Fig. 3 ▸). The obtained three-dimensional framework shows one type of channel running along the [001] direction in which the Ca^2+^ cations are located, each being coordinated by seven oxygen atoms (Fig. 4 ▸).

## Database Survey   

The formula of the title compound, CaZn_2_Fe(PO_4_)_3_, is similar to some compounds with alluaudite structures, space group *C*2/*c* or the α-CrPO_4_ structure, space group *Imma*. However, its structure is different and to our knowledge there is no known isotypic structure. Crystals of Ca*M*_2_Fe(PO_4_)_3_ (*M* = Mg, Co, Ni, Cu) compounds, which are predicted to have the same structures or isotypes are in preparation, while the structures of Sr*M*_2_Fe(PO_4_)_3_ (*M* = Co, Ni) compounds are isotypic with α-CrPO_4_ (Bouraima *et al.*, 2016 ▸; Ouaatta *et al.*, 2015 ▸). Mention may also be made of other similar compounds, for example the phosphates Na_2_Co_2_Fe(PO_4_)_3_, NaCr_2_Zn(PO_4_)_3_ and Na_1.66_Zn_1.66_Fe_1.34_(PO_4_)_3_ (Bouraima *et al.*, 2015 ▸; Souiwa *et al.*, 2015 ▸; Khmiyas *et al.*, 2015 ▸) adopting the alluaudite structure type. In conclusion, we can say that the structure of this phosphate is similar to the alluaudite structure but with lower symmetry.

## Synthesis and crystallization   

Single crystals of CaZn_2_Fe(PO_4_)_3_ were synthesized by a conventional solid-state method. Appropriate amounts of metal nitrate reagents, in the presence of H_3_PO_4_ 85 wt%, were first dissolved in deionized water in the molar ratio Ca:Zn:Fe:P = 2:2:1:3 for 24 h. Then, the resulting solution was evaporated to dryness. The powder residue was ground in an agate mortar and progressively heated in a platinum crucible at a heating rate of 141 K h^−1^ until melting occurred at 1283 K. The melted product was cooled down at a rate of 5 K h^−1^. As result of the reaction, we obtained transparent crystals corresponding to the title compound CaZn_2_Fe(PO_4_)_3_.

## Refinement   

Crystal data, data collection and structure refinement details are summarized in Table 1 ▸. The reflections (202) and (330) probably affected by the beam-stop were omitted from the refinement. The maximum and minimum electron densities in the final Fourier map are at 0.56 and 0.44 Å from Ca1 and Zn2, respectively.

## Supplementary Material

Crystal structure: contains datablock(s) I. DOI: 10.1107/S2056989016012421/pj2033sup1.cif

Structure factors: contains datablock(s) I. DOI: 10.1107/S2056989016012421/pj2033Isup2.hkl

CCDC reference: 1497218

Additional supporting information: crystallographic information; 3D view; checkCIF report

## Figures and Tables

**Table 1:** Experimental details

Crystal data	
Chemical formula	CaZn_2_Fe(PO_4_)_3_
*M*_r_	511.58
Crystal system, space group	Monoclinic, *P*2_1_/*c*
Temperature (K)	296
*a*, *b*, *c* (Å)	8.5619 (3), 15.2699 (5), 8.1190 (3)
β (°)	117.788 (2)
*V* (Å^3^)	939.06 (6)
*Z*	4
Radiation type	Mo *K*α
μ (mm^−1^)	7.72
Crystal size (mm)	0.30 × 0.26 × 0.18

Data collection	
Diffractometer	Bruker X8 *APEX*
Absorption correction	Multi-scan (*SADABS*; Krause *et al.*, 2015 ▸)
*T*_min_, *T*_max_	0.600, 0.747
No. of measured, independent and observed [*I* > 2σ(*I*)] reflections	54053, 4985, 4493
*R*_int_	0.033
(sin θ/λ)_max_ (Å^−1^)	0.859

Refinement	
*R*[*F* ^2^ > 2σ(*F* ^2^)], *wR*(*F* ^2^), *S*	0.017, 0.041, 1.04
No. of reflections	4985
No. of parameters	172
Δρ_max_, Δρ_min_ (e Å^−3^)	1.07, −0.78

Computer programs: *APEX2* and *SAINT* (Bruker, 2009 ▸), *SHELXT* (Sheldrick, 2015*a* ▸), *SHELXL2014* (Sheldrick, 2015*b* ▸), *ORTEP-3 for Windows* (Farrugia, 2012 ▸), *DIAMOND* (Brandenburg, 2006 ▸) and *publCIF* (Westrip, 2010 ▸).

## supplementary crystallographic information

### Crystal data

**Table:**

CaZn_2_Fe(PO_4_)_3_	*F*(000) = 988
*M**_r_* = 511.58	*D*_x_ = 3.618 Mg m^−^^3^
Monoclinic, *P*2_1_/*c*	Mo *K*α radiation, λ = 0.71073 Å
*a* = 8.5619 (3) Å	Cell parameters from 4985 reflections
*b* = 15.2699 (5) Å	θ = 2.7–37.6°
*c* = 8.1190 (3) Å	µ = 7.72 mm^−^^1^
β = 117.788 (2)°	*T* = 296 K
*V* = 939.06 (6) Å^3^	Block, black
*Z* = 4	0.30 × 0.26 × 0.18 mm

### Data collection

**Table:**

Bruker X8 APEX diffractometer	4985 independent reflections
Radiation source: fine-focus sealed tube	4493 reflections with *I* > 2σ(*I*)
Graphite monochromator	*R*_int_ = 0.033
φ and ω scans	θ_max_ = 37.6°, θ_min_ = 2.7°
Absorption correction: multi-scan (SADABS; Krause *et al.*, 2015)	*h* = −14→14
*T*_min_ = 0.600, *T*_max_ = 0.747	*k* = −26→26
54053 measured reflections	*l* = −10→13

### Refinement

**Table:**

Refinement on *F*^2^	172 parameters
Least-squares matrix: full	0 restraints
*R*[*F*^2^ > 2σ(*F*^2^)] = 0.017	*w* = 1/[σ^2^(*F*_o_^2^) + (0.0174*P*)^2^ + 0.7655*P*] where *P* = (*F*_o_^2^ + 2*F*_c_^2^)/3
*wR*(*F*^2^) = 0.041	(Δ/σ)_max_ = 0.003
*S* = 1.04	Δρ_max_ = 1.07 e Å^−^^3^
4985 reflections	Δρ_min_ = −0.78 e Å^−^^3^

### Special details

**Table:**

Geometry. All esds (except the esd in the dihedral angle between two l.s. planes) are estimated using the full covariance matrix. The cell esds are taken into account individually in the estimation of esds in distances, angles and torsion angles; correlations between esds in cell parameters are only used when they are defined by crystal symmetry. An approximate (isotropic) treatment of cell esds is used for estimating esds involving l.s. planes.

### Fractional atomic coordinates and isotropic or equivalent isotropic displacement parameters (Å^2^)

**Table:**

	*x*	*y*	*z*	*U*_iso_*/*U*_eq_	
Zn1	0.81685 (2)	0.73588 (2)	0.30142 (2)	0.00740 (3)	
Zn2	0.88412 (2)	0.52265 (2)	0.59417 (2)	0.01026 (3)	
Fe1	0.67075 (2)	0.49009 (2)	0.83003 (2)	0.00500 (3)	
Ca1	0.27619 (3)	0.75762 (2)	0.47919 (3)	0.01070 (4)	
P1	0.29665 (3)	0.58370 (2)	0.77261 (4)	0.00503 (4)	
P2	0.96807 (3)	0.62244 (2)	0.09438 (4)	0.00499 (4)	
P3	0.60325 (3)	0.64096 (2)	0.49014 (4)	0.00491 (4)	
O1	0.29942 (13)	0.67932 (6)	0.72349 (13)	0.01271 (15)	
O2	0.30273 (12)	0.58596 (6)	0.96463 (12)	0.01006 (14)	
O3	0.12207 (10)	0.54161 (6)	0.62684 (12)	0.01114 (15)	
O4	0.43948 (11)	0.52893 (6)	0.76475 (14)	0.01312 (16)	
O5	0.77926 (10)	0.58832 (5)	0.00379 (12)	0.00895 (13)	
O6	1.00136 (11)	0.67493 (6)	−0.04745 (13)	0.01045 (14)	
O7	1.00262 (11)	0.68143 (6)	0.26134 (13)	0.01055 (14)	
O8	1.09932 (10)	0.54481 (5)	0.17408 (12)	0.00724 (13)	
O9	0.75426 (11)	0.65280 (6)	0.43941 (13)	0.01160 (15)	
O10	0.59377 (10)	0.71947 (6)	0.60355 (12)	0.00974 (14)	
O11	0.66602 (11)	0.55677 (5)	0.61102 (12)	0.00822 (13)	
O12	0.41993 (10)	0.62799 (6)	0.32620 (12)	0.01012 (14)	

### Atomic displacement parameters (Å^2^)

**Table:**

	*U*^11^	*U*^22^	*U*^33^	*U*^12^	*U*^13^	*U*^23^
Zn1	0.00645 (5)	0.00817 (5)	0.00801 (6)	0.00013 (4)	0.00374 (4)	0.00104 (4)
Zn2	0.00760 (5)	0.01471 (6)	0.01073 (6)	0.00042 (4)	0.00616 (4)	−0.00080 (5)
Fe1	0.00454 (5)	0.00602 (6)	0.00488 (6)	0.00044 (4)	0.00256 (4)	0.00061 (4)
Ca1	0.01029 (8)	0.01095 (9)	0.01178 (10)	0.00175 (6)	0.00591 (7)	0.00601 (7)
P1	0.00467 (9)	0.00607 (10)	0.00452 (10)	0.00011 (7)	0.00229 (8)	−0.00022 (8)
P2	0.00441 (9)	0.00482 (10)	0.00581 (11)	0.00038 (7)	0.00243 (8)	−0.00009 (8)
P3	0.00448 (9)	0.00500 (10)	0.00483 (10)	0.00004 (7)	0.00182 (8)	0.00031 (8)
O1	0.0222 (4)	0.0076 (3)	0.0109 (4)	−0.0001 (3)	0.0098 (3)	0.0019 (3)
O2	0.0169 (3)	0.0093 (3)	0.0063 (3)	0.0009 (3)	0.0073 (3)	0.0011 (3)
O3	0.0051 (3)	0.0176 (4)	0.0102 (4)	−0.0030 (3)	0.0030 (3)	−0.0063 (3)
O4	0.0065 (3)	0.0172 (4)	0.0157 (4)	0.0026 (3)	0.0052 (3)	−0.0045 (3)
O5	0.0056 (3)	0.0100 (3)	0.0101 (3)	−0.0016 (2)	0.0028 (2)	−0.0034 (3)
O6	0.0097 (3)	0.0107 (3)	0.0132 (4)	0.0029 (3)	0.0072 (3)	0.0061 (3)
O7	0.0079 (3)	0.0122 (3)	0.0115 (4)	−0.0005 (2)	0.0045 (3)	−0.0062 (3)
O8	0.0065 (3)	0.0073 (3)	0.0088 (3)	0.0026 (2)	0.0043 (2)	0.0020 (2)
O9	0.0111 (3)	0.0118 (3)	0.0161 (4)	0.0008 (3)	0.0098 (3)	0.0042 (3)
O10	0.0076 (3)	0.0091 (3)	0.0103 (3)	0.0006 (2)	0.0024 (3)	−0.0040 (3)
O11	0.0100 (3)	0.0080 (3)	0.0082 (3)	0.0028 (2)	0.0055 (3)	0.0039 (3)
O12	0.0068 (3)	0.0086 (3)	0.0097 (3)	−0.0002 (2)	−0.0006 (3)	−0.0020 (3)

### Geometric parameters (Å, º)

**Table:**

Zn1—O9	1.9266 (9)	Ca1—O2^i^	2.4075 (9)
Zn1—O7	1.9518 (8)	Ca1—O7^viii^	2.4709 (9)
Zn1—O10^i^	1.9578 (8)	Ca1—O6^ix^	2.4840 (9)
Zn1—O6^ii^	2.0120 (8)	Ca1—O10	2.4885 (8)
Zn2—O3^iii^	1.9496 (8)	Ca1—O12	2.8984 (9)
Zn2—O11	2.0038 (8)	P1—O4	1.5073 (9)
Zn2—O3^iv^	2.0241 (9)	P1—O1	1.5166 (9)
Zn2—O8^v^	2.0911 (8)	P1—O2	1.5358 (9)
Zn2—O9	2.3371 (9)	P1—O3	1.5487 (8)
Fe1—O4	1.8908 (8)	P2—O5	1.5222 (8)
Fe1—O2^vi^	1.9561 (9)	P2—O6	1.5348 (9)
Fe1—O5^vii^	1.9700 (8)	P2—O7	1.5371 (9)
Fe1—O11	2.0330 (8)	P2—O8	1.5519 (8)
Fe1—O8^v^	2.0547 (8)	P3—O12	1.5253 (8)
Fe1—O12^iv^	2.1318 (8)	P3—O10	1.5365 (9)
Ca1—O1	2.2439 (9)	P3—O9	1.5396 (9)
Ca1—O1^i^	2.3795 (10)	P3—O11	1.5534 (8)
			
O9—Zn1—O7	106.60 (4)	O2^i^—Ca1—O6^ix^	72.04 (3)
O9—Zn1—O10^i^	106.09 (4)	O7^viii^—Ca1—O6^ix^	65.76 (3)
O7—Zn1—O10^i^	124.80 (4)	O1—Ca1—O10	83.50 (3)
O9—Zn1—O6^ii^	116.31 (4)	O1^i^—Ca1—O10	85.92 (3)
O7—Zn1—O6^ii^	85.46 (3)	O2^i^—Ca1—O10	98.16 (3)
O10^i^—Zn1—O6^ii^	116.93 (4)	O7^viii^—Ca1—O10	132.22 (3)
O3^iii^—Zn2—O11	154.16 (4)	O6^ix^—Ca1—O10	160.73 (3)
O3^iii^—Zn2—O3^iv^	77.67 (4)	O1—Ca1—O12	97.73 (3)
O11—Zn2—O3^iv^	123.13 (3)	O1^i^—Ca1—O12	71.19 (3)
O3^iii^—Zn2—O8^v^	108.82 (4)	O2^i^—Ca1—O12	126.00 (3)
O11—Zn2—O8^v^	75.00 (3)	O7^viii^—Ca1—O12	79.74 (3)
O3^iv^—Zn2—O8^v^	121.44 (4)	O6^ix^—Ca1—O12	145.10 (3)
O3^iii^—Zn2—O9	98.73 (4)	O10—Ca1—O12	53.99 (3)
O11—Zn2—O9	65.84 (3)	O1—Ca1—O12^ii^	69.93 (3)
O3^iv^—Zn2—O9	97.26 (4)	O1^i^—Ca1—O12^ii^	114.39 (3)
O8^v^—Zn2—O9	135.92 (3)	O2^i^—Ca1—O12^ii^	57.96 (3)
O4—Fe1—O2^vi^	96.63 (4)	O7^viii^—Ca1—O12^ii^	140.52 (3)
O4—Fe1—O5^vii^	92.55 (4)	O6^ix^—Ca1—O12^ii^	78.49 (3)
O2^vi^—Fe1—O5^vii^	90.75 (4)	O10—Ca1—O12^ii^	82.24 (3)
O4—Fe1—O11	90.37 (4)	O12—Ca1—O12^ii^	135.98 (3)
O2^vi^—Fe1—O11	171.82 (3)	O4—P1—O1	114.24 (6)
O5^vii^—Fe1—O11	93.18 (4)	O4—P1—O2	114.28 (5)
O4—Fe1—O8^v^	164.34 (4)	O1—P1—O2	104.36 (5)
O2^vi^—Fe1—O8^v^	97.38 (3)	O4—P1—O3	104.50 (5)
O5^vii^—Fe1—O8^v^	94.22 (3)	O1—P1—O3	109.05 (5)
O11—Fe1—O8^v^	75.18 (3)	O2—P1—O3	110.42 (5)
O4—Fe1—O12^iv^	93.16 (4)	O5—P2—O6	110.07 (5)
O2^vi^—Fe1—O12^iv^	82.55 (4)	O5—P2—O7	110.55 (5)
O5^vii^—Fe1—O12^iv^	171.65 (3)	O6—P2—O7	109.20 (5)
O11—Fe1—O12^iv^	92.86 (4)	O5—P2—O8	109.84 (5)
O8^v^—Fe1—O12^iv^	81.77 (3)	O6—P2—O8	111.11 (5)
O1—Ca1—O1^i^	167.91 (4)	O7—P2—O8	106.00 (5)
O1—Ca1—O2^i^	126.91 (3)	O12—P3—O10	107.69 (5)
O1^i^—Ca1—O2^i^	60.49 (3)	O12—P3—O9	115.63 (5)
O1—Ca1—O7^viii^	92.62 (3)	O10—P3—O9	110.75 (5)
O1^i^—Ca1—O7^viii^	90.17 (3)	O12—P3—O11	110.86 (5)
O2^i^—Ca1—O7^viii^	120.77 (3)	O10—P3—O11	111.50 (5)
O1—Ca1—O6^ix^	89.31 (3)	O9—P3—O11	100.36 (5)
O1^i^—Ca1—O6^ix^	102.55 (3)		

Symmetry codes: (i) *x*, −*y*+3/2, *z*−1/2; (ii) *x*, −*y*+3/2, *z*+1/2; (iii) *x*+1, *y*, *z*; (iv) −*x*+1, −*y*+1, −*z*+1; (v) −*x*+2, −*y*+1, −*z*+1; (vi) −*x*+1, −*y*+1, −*z*+2; (vii) *x*, *y*, *z*+1; (viii) *x*−1, *y*, *z*; (ix) *x*−1, −*y*+3/2, *z*+1/2.
